# News Media and the Influence of the Alcohol Industry: An Analysis of Media Coverage of Alcohol Warning Labels With a Cancer Message in Canada and Ireland

**DOI:** 10.15288/jsad.2020.81.273

**Published:** 2020-05-03

**Authors:** Kate Vallance, Alexandria Vincent, Nour Schoueri-mychasiw, Tim Stockwell, David Hammond, Thomas K. Greenfield, Jonathan McGavock, Erin Hobin

**Affiliations:** ^a^ Canadian Institute for Substance Use Research, University of Victoria, Victoria, British Columbia, Canada; ^b^ University of Guelph, Guelph, Ontario, Canada; ^c^ Health Promotion, Chronic Disease and Injury Prevention, Public Health Ontario, Toronto, Ontario, Canada; ^d^ School of Public Health & Health Systems, University of Waterloo, Waterloo, Ontario, Canada; ^e^ Alcohol Research Group, Public Health Institute, Emeryville, California, United States; ^f^ Department of Pediatrics and Child Health, Faculty of Health Sciences, University of Manitoba, Winnipeg, Manitoba, Canada; ^g^ Dalla Lana School of Public Health, University of Toronto, Toronto, Ontario, Canada

## Abstract

**Objective:**

Media coverage of alcohol-related policy measures can influence public debate and is often more aligned with interests of the alcohol industry than public health. The purpose of this study was to examine the framing of news coverage of alcohol warning label (AWL) initiatives that included a cancer message on alcohol containers in two different countries. Policy contexts and industry perspectives were also evaluated.

**Method:**

We identified and systematically reviewed news articles published between 2017–2019 covering an AWL academic study in Yukon, Canada, and labeling provisions in a Public Health (Alcohol) Bill in Ireland. Both included a cancer message. News stories were coded for media type and topic slant; inclusion of alcohol industry perspectives was examined using content analysis.

**Results:**

Overall, 68.4% of media articles covering the Yukon Study (*n* = 38) and 18.9% covering the Ireland Bill (*n* = 37) were supportive of AWLs with a cancer message. The majority of articles in both sites presented alcohol industry perspectives (Yukon, 65.8%; Ireland, 86.5%), and industry arguments opposing AWLs were similar across both contexts. In articles with statements from industry representatives, the label message was frequently disputed by distorting or denying the evidence that alcohol causes cancer (*n* = 33/43).

**Conclusions:**

News coverage of AWLs with a cancer message was more supportive in Canada than Ireland, where alcohol industry perspectives were consistently foregrounded. Industry arguments opposing the cancer label bore similarities across contexts, often distorting or denying the evidence. Increasing awareness of industry messaging strategies may generate more critical coverage of industry lobbying activities and increase public support for alcohol policies. (*J. Stud. Alcohol Drugs, 81,* 273–283, 2020)

Alcohol causes 3.3 million deaths globally each year, with a substantial proportion attributable to cancer, producing significant societal harms and costs to governments and health systems worldwide (Baan et al., 2007; Bagnardi et al., 2015; Burton & Sheron, 2018; International Agency for Research on Cancer, 2010; Klein et al., 2019; Praud et al., 2016; World Health Organization, 2018). Recent projections show an increase in alcohol consumption internationally of up to 17% over the next decade, which will not only affect people consuming alcohol but also increase the exposure to harm of those around them (Karriker-Jaffe et al., 2018; Manthey et al., 2019). There are several population-level control measures (also known as “best buys”; e.g., increased alcohol pricing and taxation, reductions in physical availability, restrictions on marketing and advertising, and impaired driving countermeasures) that can be used as a response, are cost-effective, and have the strongest evidence for reducing alcohol-related harms, including cancer (Alattas et al., 2020; Chisholm et al., 2018; Foster et al., 2019; World Health Organization, 2013).

Governments often face political barriers to introducing alcohol control policies because of public pushback and opposition from powerful alcohol industry lobby groups (Hope, 2006; Li et al., 2017; Moskalewicz et al., 2013; Pechey et al., 2014). Lobbying from industry groups highlights an inherent conflict as a substantial portion of their sector’s profits rely on harmful patterns of consumption and low consumer knowledge of cancer risk linked to alcohol (Bhattacharya et al., 2018; Casswell et al., 2016; Connor, 2017). Importantly, increasing public awareness of alcohol-related health risks such as cancer has been shown to improve public support for more restrictive policies (Bates et al., 2018; Buykx et al., 2015; Martin et al., 2018; Weerasinghe et al., 2020). Alcohol warning labels (AWLs) offer one avenue for providing this type of information directly to alcohol consumers—particularly higher volume consumers (Greenfield, 1997). Furthermore, AWLs, including those with a cancer warning, have evidence of public support (Hobin et al., 2018; Maynard et al., 2018; Miller et al., 2016; Pettigrew et al., 2014; Vallance et al., 2018).

News media is a strong influencer of public debate and can often shape public opinion and affect policymaking (Macnamara, 2005; McCombs & Shaw, 1972, 1993). This is especially true in relation to government efforts to introduce control measures designed to improve public health. There is a long history of the tobacco industry (Durrant et al., 2003; Miller et al., 2018)—and, more recently, the alcohol and sugar-sweetened beverage industries (Hilton et al., 2019)—using the media as a platform to promote their vested interests at the expense of public health interventions. The unhealthy commodity industries more generally have shown great adeptness at having their perspectives regularly included in news media coverage, often with the effect of slanting articles in their favor (Azar at al., 2014; Katikireddi & Hilton, 2015; Mercille, 2017). Researchers have identified a “playbook” of scripts and messaging strategies circulated by the unhealthy commodity industries through a variety of media channels—including industry trade magazines and public-facing industry-funded social aspects public relations organizations—designed to influence public opinion, manipulate the news agenda, and influence policymaking (Hilton et al., 2019; Lim et al., 2019; Maani Hessari et al., 2019; McCambridge et al., 2018; Mercille, 2017; Petticrew et al., 2017, 2018a, 2018b; Pettigrew et al., 2018).

The unhealthy commodity industries messaging strategies used by the alcohol industry via these different platforms have been shown to disseminate selective or false evidence and information designed to confuse or misdirect public understanding of health issues or minimize the perceived risk of their product (Lim et al., 2019; Maani Hessari et al., 2019; Petticrew et al., 2017, 2018a, 2018b; Pettigrew et al., 2018). A prime example is the alcohol industry’s consistent misrepresentation of the established scientific evidence linking alcohol consumption with an increased risk of cancer. This misrepresentation often takes the form of disputing or denying the link; claiming the cancer risk is related only to heavier consumption; stating the evidence is too complex, insufficient, or debatable; and confusing the issue by claiming alcohol’s protective effects (Hilton et al., 2019; Petticrew et al., 2017, 2018a, 2018b).

Alcohol industry messaging strategies specific to news media coverage—many of which were noted during the introduction of minimum unit pricing in Scotland—include questioning the legality of or overemphasizing the economic harms resulting from implementation of proposed control policies and making unsupported claims that the measures are ineffective or are not based on sound evidence (Hilton et al., 2014; Katikireddi & Hilton, 2015; Mercille, 2017). Further, industry media statements will often protest their exclusion from the design of public health interventions and highlight their involvement in voluntary initiatives and corporate social responsibility activities, such as campaigns with vague “responsible drinking” messages. Many will also offer suggestions of other “more appropriate” measures, most of which are industry friendly and/or shown to have little or no impact on alcohol consumption and harms (Babor et al., 2018; Fogarty & Chapman, 2012; Hilton et al., 2014; Katikireddi & Hilton, 2015; Mercille, 2017; Savell et al., 2016).

Although there is a growing body of research identifying tactics commonly used across the unhealthy commodity industries and in response to specific alcohol control measures such as alcohol pricing and marketing and advertising restrictions, to our knowledge there have been limited investigations of news media coverage of AWL policy initiatives (Lemmens et al., 1999) and none specific to AWLs with a cancer message. The purpose of this study was to examine news media coverage of alcohol labeling initiatives that included a cancer warning in Canada and Ireland and analyze the perspectives of the alcohol industry that were included in the articles. Specifically, objectives were to (a) compare the topic slant of mass media news coverage of alcohol industry interference with a cancer warning label that formed part of an academic study in Yukon, Canada (“the Yukon Study”), with coverage of cancer warning label provisions included in Ireland’s Public Health (Alcohol) Bill (“the Ireland Bill”) and (b) identify similarities and/or differences in the inclusion and content of the alcohol industry’s response to AWLs with a cancer message within media coverage across both contexts.

## Method

### Context

#### Academic Study in Yukon, Canada (“the Yukon Study”).

The authors of this article implemented a study funded by the federal health institution in Canada designed to test the effectiveness of three new evidence-informed AWLs that featured (a) a health message stating alcohol can cause cancer, including breast and colon cancers, (b) national low-risk drinking guidelines, and (c) standard drink information. The new labels formed part of a quasi-experimental study in Whitehorse, Yukon (intervention site) and Yellowknife, Northwest Territories (comparison site), both located in northern Canada (see Vallance et al., 2020, for full study protocol). Both sites had already been applying post-manufacturer text-based AWLs by local directive since 1991 cautioning about the risks of drinking during pregnancy; the Northwest Territories label carries an additional warning similar to that of the mandatory US label (Alcoholic Beverage Health Warning Statement, 2008) about impaired driving and general health risks. The three new rotating labels were to be applied to alcohol containers in the only government-run liquor store in the intervention site for an 8-month period, whereas the two liquor stores in the comparison site continued usual labeling practices. (Images of all labels can be viewed at alcohollabels.cisur.ca.) The new labels were launched on November 20, 2017, but halted on December 19, 2017, because of alcohol industry interference (Wilt, 2018). The territorial government agreed to resume the study in February 2018 on the condition that the cancer warning label be permanently removed from rotation to avoid potential litigation by the alcohol industry (Joannou, 2018).

### Public Health (Alcohol) Bill in Ireland (“the Ireland Bill”)

The Public Health (Alcohol) Bill was first introduced into Irish parliament in 2015 as a public health measure to address high rates of alcohol consumption and related harm in the country. The Ireland Bill contained a number of alcohol control provisions, including minimum pricing; structural separation of alcohol from other products in stores; bans on alcohol sponsorship; restrictions on marketing and advertising; and mandatory health messaging on alcohol containers, including a cancer warning (Murray, 2017). The Ireland Bill’s progress through parliament was delayed by 2 years as a result of intense lobbying from the alcohol industry. A revised version of the Ireland Bill was passed through the second stage in the lower house of parliament in 2017, dropping the provisions around sponsorship and amending some of the marketing and advertising restrictions (Mercille, 2017). The Ireland Bill passed through the committee stage in the upper house of parliament in March 2018 and amendments were proposed in June 2018, including the removal of the labeling provisions, and discussed in the upper house assembly in September of that year. The Ireland Bill reached the final stage of parliament and was passed into law in October 2018. A commencement order for some of the provisions (not including labeling) was signed on November 1, 2018.

### Search parameters and record selection

The search period for news coverage of AWLs related to the Yukon Study was September 1, 2017, to August 15, 2018, covering 3 months before the media release announcing the launch of the new AWLs and 6 months after the media release announcing the study’s resumption in February 2018. The search period for news coverage of AWLs related to the Ireland Bill was April 16, 2018, to April 30, 2019, covering the 6 months before and after the Ireland Bill was signed into law in October 2018. These timeframes were selected in accordance with the main media events connected to both contexts; each site had an approximately 1-year search period to ensure feasibility of the study parameters.

**Figure 1.**
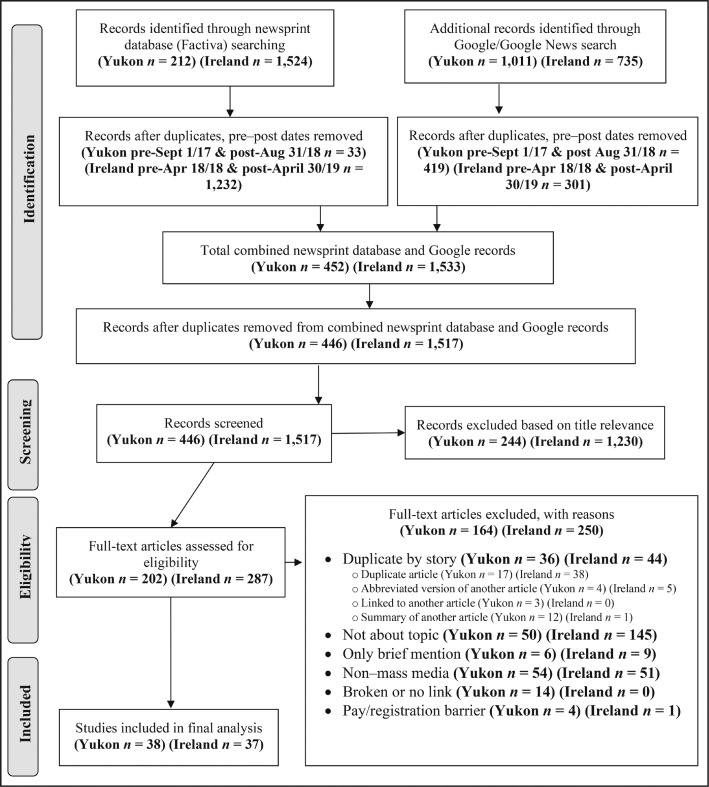
PRISMA article eligibility screening flow diagram—Yukon Study & Ireland Bill

Six sets of search strings were developed to identify relevant media coverage of both the Yukon Study and the Ireland Bill (see search strings in Appendix A). (The appendices appear as online-only addenda to this article on the journal’s website.) Separate searches for each string were run in Factiva, an electronic media database with comprehensive full-text coverage of both Canadian and European Union sources. Equivalent search strings were run in the web-based search engines Google and Google News, capturing the first 100 hits for each string; no date limits were set because web-based search engines can indicate date only in year segments (e.g., in the past year). A systematic strategy was used to manually determine eligible database and web-based records for inclusion based on PRISMA (Preferred Reporting Items for Systematic Reviews and Meta-Analyses) guidelines (Moher et al., 2009) (Figure 1). All remaining records were then screened for relevance based on title and subsequently assessed for eligibility with a full-text review. The following records were excluded: duplicates by story, articles that did not qualify as mass media or text-based news sources (as determined by the research team), articles that did not/only briefly mentioned the Yukon Study or the labeling provisions of the Ireland Bill, and articles with broken or missing hyperlinks or with registration or paywall barriers.

### Coding and analysis

The coding structure to identify media type and topic slant for this analysis was developed based on previous studies (Azar et al., 2014; Mercille, 2017; Miller et al., 2018). Media type was coded by article publication source and included the following categories: (a) hard news, (b) web story, (c) commentary, and (d) blog (see Table 1 for media type definitions). Two of the authors (K.V. and A.V.) coded and discussed an initial subsample of articles by media type and subsequently independently coded the full sample with no divergences between coders. Topic slant was coded based on whether the stories’ viewpoints were predominantly neutral, supportive, or opposed—or a mix of both supportive and opposed—toward AWLs with a cancer message included in the Yukon Study and the Ireland Bill (see Table 1 for code definitions). The same two authors coded an initial subsample of articles for topic slant (*n* = 8), resulting in a Cohen’s κ score of .79, indicative of substantial agreement among coders (McHugh, 2012). Divergences were discussed with the team to achieve consensus, and the codebook was refined before final coding. Using the refined codebook, two of the authors (K.V. and N.S.M.) independently coded the full sample (*n* = 75), resulting in a Cohen’s κ score of .90, representing near perfect agreement between coders (McHugh, 2012).

**Table 1.**
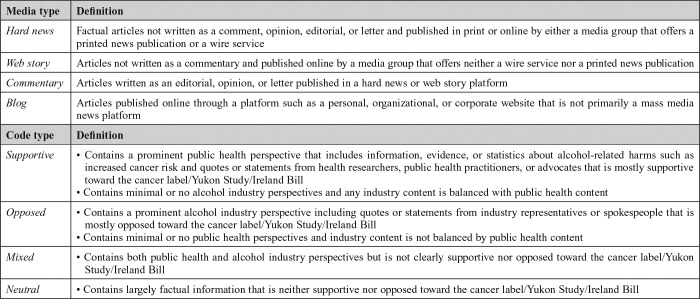
Definitions of media type and topic slant codes

**Media type**	**Definition**
*Hard news*	Factual articles not written as a comment, opinion, editorial, or letter and published in print or online by either a media group that offers a printed news publication or a wire service
*Web story*	Articles not written as a commentary and published online by a media group that offers neither a wire service nor a printed news publication
*Commentary*	Articles written as an editorial, opinion, or letter published in a hard news or web story platform
*Blog*	Articles published online through a platform such as a personal, organizational, or corporate website that is not primarily a mass media news platform

**Code type**	**Definition**
*Supportive*	• Contains a prominent public health perspective that includes information, evidence, or statistics about alcohol-related harms such as increased cancer risk and quotes or statements from health researchers, public health practitioners, or advocates that is mostly supportive toward the cancer label/Yukon Study/Ireland Bill
	• Contains minimal or no alcohol industry perspectives and any industry content is balanced with public health content
*Opposed*	• Contains a prominent alcohol industry perspective including quotes or statements from industry representatives or spokespeople that is mostly opposed toward the cancer label/Yukon Study/Ireland Bill
	• Contains minimal or no public health perspectives and industry content is not balanced by public health content
*Mixed*	• Contains both public health and alcohol industry perspectives but is not clearly supportive nor opposed toward the cancer label/Yukon Study/Ireland Bill
*Neutral*	• Contains largely factual information that is neither supportive nor opposed toward the cancer label/Yukon Study/Ireland Bill

The number of articles was calculated by media type and topic slant, and the coded articles were grouped by calendar month for each of the two sites. The number of articles containing alcohol industry perspectives was then calculated, and content analysis (Elo & Kyngäs, 2008) was conducted to inductively identify, group, and enumerate the most prominent alcohol industry arguments included in the news coverage. Industry *arguments* were defined as statements or quotes related to the AWLs attributed to specific alcohol industry representatives and spokespeople or related viewpoints attributed to the alcohol industry more broadly. In some cases, articles included multiple distinct industry statements or viewpoints that each contained multiple distinct arguments within; each of these arguments was recorded individually. The number of articles containing *direct* statements or quotes from industry representatives or spokespeople was also calculated, and the proportion of those disputing the evidence linking alcohol and cancer was identified.

## Results

### Type and overall topic slant of media articles

**Figure 2.**
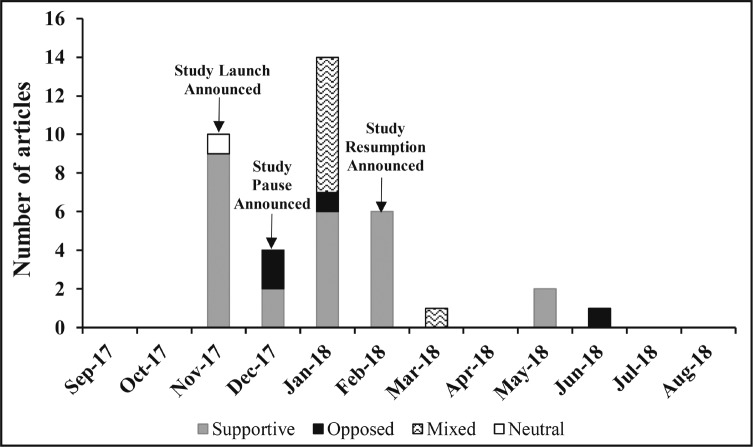
Topic slant of media articles 2017–2018, Yukon Study (n = 38)

We identified 38 articles eligible for inclusion related to the Yukon Study (see Appendix B for full list of articles) during the set timeframe. Of those, two thirds (*n* = 25) were supportive of the Yukon Study and the cancer warning; more than half (*n* = 21) were published in hard news sources (Appendix C). The publication dates of articles related to the Yukon Study ranged from November 22, 2017, to June 21, 2018, with the majority published in January 2018 following the announcement that application of the study intervention labels at the liquor store had been halted as a result of alcohol industry pressure (Figure 2).

**Figure 3.**
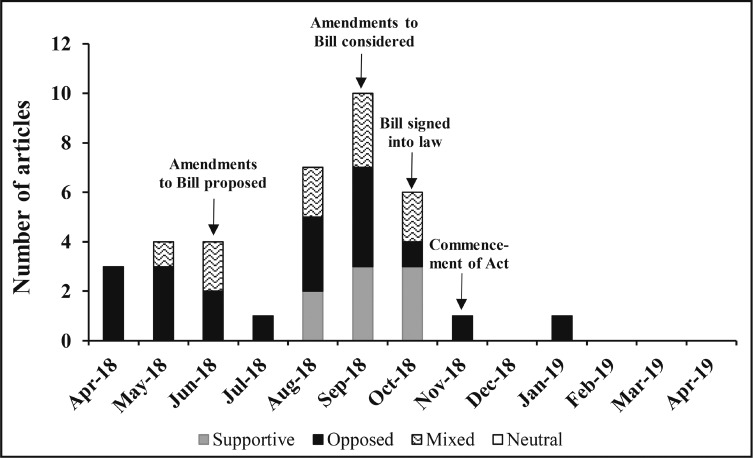
Topic slant of media articles 2018–2019, Ireland Bill (n = 37)

There were 37 eligible articles related to the Ireland Bill during the set timeframe (Appendix B). Of those, less than a quarter (*n* = 8) were supportive of the labeling provisions in the Bill and the cancer warning; more than half (*n* = 23) were published in hard news sources (Appendix C). The publication dates of the Ireland Bill articles ranged from April 19, 2018, to January 18, 2019, with nearly half (*n* = 17) published between August and September 2018 leading up to the proposed labeling amendments (including dropping the labeling provision) being debated in the parliamentary assembly (Figure 3).

### Inclusion of alcohol industry perspectives in media articles

Two thirds (*n* = 25) of the Yukon Study media coverage and more than three quarters (*n* = 32) of the articles covering the Ireland Bill included the perspectives of the alcohol industry. The main industry actors represented in the Yukon Study coverage were the heads of Canada’s three main national alcohol industry trade lobby associations: Beer Canada, Spirits Canada, and the Canadian Vintner’s Association. The industry actors represented in the Ireland Bill media coverage were predominantly representatives from lobby groups, including the Alcohol Beverage Foundation of Ireland (ABFI) and the Irish Whiskey Association, as well as a variety of spokespeople from independent breweries and distilleries. Just over one third (*n* = 14) of the Yukon Study media articles and three quarters (*n* = 29) of the Ireland Bill articles contained *direct* statements or quotes from alcohol industry representatives or spokespeople.

### Main industry arguments opposing AWLs

#### Distorting the evidence and cancer denialism.

The most frequent alcohol industry argument opposing AWL common to the news coverage of both the Yukon Study and the Ireland Bill was to attack the content and validity of the cancer label message itself (Table 2). Media articles consistently contained industry perspectives that distorted, downplayed, and otherwise obfuscated the evidence linking alcohol and cancer. In many instances, industry arguments claimed the cancer warning was inaccurate, unproven, and based on false or unsound evidence. Arguments also frequently highlighted risk factors for cancer aside from alcohol, stated that the alcohol–cancer evidence was “too complex for a single label,” and alluded to alcohol’s health benefits. A number of these arguments were often contained within a single statement or quote.

**Table 2.**
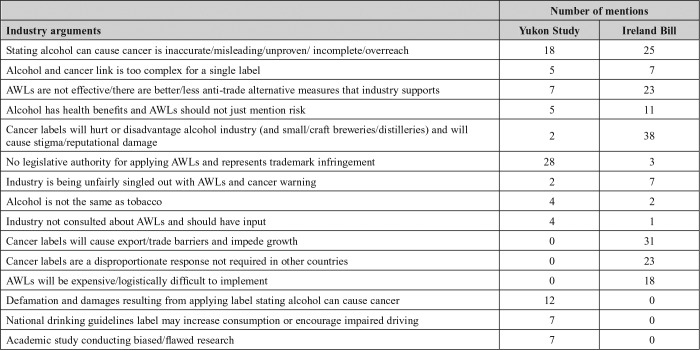
Industry arguments opposing alcohol warning labels (AWLs) and the cancer warning mentioned in news articles

Industry arguments	Yukon Study	Ireland Bill
	Number of mentions	
Stating alcohol can cause cancer is inaccurate/misleading/unproven/incomplete/overreach	18	25
Alcohol and cancer link is too complex for a single label	5	7
AWLs are not effective/there are better/less anti-trade alternative measures that industry supports	7	23
Alcohol has health benefits and AWLs should not just mention risk	5	11
Cancer labels will hurt or disadvantage alcohol industry (and small/craft breweries/distilleries) and will cause stigma/reputational damage	2	38
No legislative authority for applying AWLs and represents trademark infringement	28	3
Industry is being unfairly singled out with AWLs and cancer warning	2	7
Alcohol is not the same as tobacco	4	2
Industry not consulted about AWLs and should have input	4	1
Cancer labels will cause export/trade barriers and impede growth	0	31
Cancer labels are a disproportionate response not required in other countries	0	23
AWLs will be expensive/logistically difficult to implement	0	18
Defamation and damages resulting from applying label stating alcohol can cause cancer	12	0
National drinking guidelines label may increase consumption or encourage impaired driving	7	0
Academic study conducting biased/flawed research	7	0

Of the 43 articles that contained *direct* statements or quotes attributed to alcohol industry representatives or spokespeople, nearly half distorted (*n* = 19/43) and one third denied (*n* = 14/43) the scientific evidence behind the cancer warning label. Denialism of the alcohol–cancer evidence is highlighted by comments from the president of Beer Canada who stated that: “. . . the (cancer) label they chose to use is inaccurate and misleading” and “. . . to claim that alcohol causes cancer, or can cause cancer, is not accurate . . .” (*National Post,* January 2, 2018). The president of Spirits Canada also indicated that “the content of the label that reads ‘alcohol can cause cancer, including breast and coloncancers’ is scientifically debatable . . .” and that “we’re not very happy with the presentation that drinking alcohol in moderate or light amounts causes cancer. There’s really no evidence of causality; there’s some correlation evidence” (*Whitehorse Star,* January 5, 2018).

Further distortion and denialism of the evidence is illustrated by ABFI’s chief who was quoted as saying: “. . . We are not in support of providing consumers with inaccurate and misleading information that causes confusion and damages business. The association between alcohol and cancer is complex and cannot be adequately explained in a single warning label. One drink does not give you cancer, so it is inaccurate to place a blunt warning on alcohol products to say that alcohol causes cancer . . .” (*Irish Sun,* June 19, 2018). The Irish industry lobby group’s chief later stated that “The scientific evidence certainly doesn’t warrant the direct link between alcohol and cancer . . . on the contrary low consumption was beneficial to health” (*Euractiv,* October 11, 2018).

### Offering “better” alternatives to AWL initiatives

Industry arguments opposing the AWLs in the media coverage of both the Yukon Study and the Ireland Bill also frequently mentioned that there were other more effective and less anti-trade control measures that could be implemented or that industry had already voluntarily initiated (Table 2). The president of Spirits Canada stated that his group: “. . . supported the creation of low-risk drinking guidelines and there are better ways to communicate the risks associated with heavy drinking, including through advertising campaigns and alcohol sellers having conversations with their customers” (*Canadian Press,* January 3, 2018). Similarly, the head of the Irish Whiskey Association suggested that “alternative measures that are less harmful to trade, such as public information campaigns, education initiatives and specific targeted interventions, should be used to help tackle misuse” (*Drinks Industry Ireland,* September 4, 2018). ABFI’s Chief further argued: “While we as an industry support the objectives of the Alcohol Bill to tackle harmful and underage drinking in Ireland, we are opposed to cancer warning labels and believe that the objectives of the Alcohol Bill could be achieved through more effective and less trade-restrictive means” (*The Irish Times,* August 2, 2018).

### Questioning the legality of the AWLs and the design of the Yukon Study

By far, the most frequent industry arguments specific to the Yukon Study news coverage focused on alcohol industry concerns around the territory’s legislative authority for placing “nonauthorized” AWLs on containers, potential trademark infringement resulting from AWLs, and defamation and damages related to the cancer label message. Other arguments centered on assertions that the Yukon Study team was conducting biased and “fatally flawed” research and on the industry’s objection to not being consulted about the study or the AWL content and design (Table 2). The industry’s expectation to have input was highlighted by comments from the president of the Canadian Vintner’s Association who indicated, “There isn’t opposition to labelling *per say* [sic], but the way that the labels were selected without consultation with industry and without consultation with the National Alcohol Strategy Advisory Committee that has expertise to ensure that the labels are accurate, and beneficial” (*Whitehorse Star,* January 5, 2018).

### Putting economic interests and the Irish alcohol industry’s reputation first

In Ireland, the industry’s arguments against the cancer warning label focused heavily on a wide range of serious economic trade barriers they felt the cancer labels would create within the European Commission and across international markets. Additional arguments claimed that introducing a cancer warning label would severely hurt or disadvantage the Irish alcohol industry, would make Ireland a “global pariah,” and was a disproportionate response “required nowhere else in the world” (Table 2). As the head of the Irish Whiskey Association commented in one article, “cancer warnings on alcohol products . . . will cause serious harm to the reputation of the Irish drinks industry . . . yet is unlikely to do anything to combat alcohol misuse or harmful drinking” (*Drinks Industry Ireland,* September 4, 2018). He later stated that the cancer label “imposes an internationally unprecedented stigma on Irish whiskey . . . which our competitors, the Scotch and Bourbon whiskey tourism industry, don’t face” (*Irish Examiner,* January 14, 2019).

## Discussion

This article explored media coverage of two AWL initiatives that included a cancer message and examined the alcohol industry perspectives presented within the news articles published across two geographical contexts. To our knowledge, very few studies have analyzed news coverage of AWLs, with this being the first specific to cancer warning labels. Media coverage of the Yukon Study in Canada and subsequent alcohol industry interference was largely supportive of the AWL study that included a cancer label. In contrast, news coverage of the cancer labeling provisions proposed in Ireland’s Public Health (Alcohol) Bill was primarily negative and consistently foregrounded alcohol industry perspectives. There was only one article across both sites without a topic slant that was opposed, supportive, or a combination of both, suggesting that cancer labels represent a contentious policy measure that has the potential to elicit a strong and targeted media response from the alcohol industry across different geographical and policy contexts.

Perhaps the most important finding in this study is that alcohol industry arguments opposing the cancer warning label across news articles in both countries consistently attempted to undermine and obfuscate the well-established scientific evidence causally linking alcohol consumption to increased cancer risk. This finding is consistent with previous investigations of industry messaging shown to misrepresent the evidence around alcohol and cancer (Maani Hessari & Petticrew, 2018; Maani Hessari et al., 2019; Petticrew et al., 2018a, 2018b; Pettigrew et al., 2018) and use the complexity argument as a strategy to influence public perception and oppose policy measures (Hilton et al., 2019; Pettigrew et al., 2017). The blatant cancer denialism and distortion of the evidence are particularly striking given the breadth of the industry voices included in mainstream media and the documented dissemination of false and misleading information to the public by industry-funded social aspects public relations organizations in Canada and Ireland (Lim et al., 2019; Petticrew et al., 2018a).

Overall, the structure, format, and content of the arguments used by the alcohol industry to oppose AWLs and the cancer label in media coverage of the Yukon Study and the Ireland Bill was consistent with previously identified scripts that form part of the cross-industry playbook of messaging strategies (Hilton et al., 2019; Petticrew et al., 2017). Arguments often incorporated many rationales into a single statement and were repeated consistently, regardless of their veracity, which is a tactic of persuasion identified in media coverage of other alcohol control measures such as minimum unit pricing and marketing and advertising restrictions (Hilton et al., 2014; Katikireddi & Hilton, 2015; Mercille, 2017). Statements by industry representatives that attempted to redirect media messaging away from the AWL measures by claiming they were already making efforts to address harmful alcohol use and suggesting policy alternatives and responses deemed more effective and appropriate were also consistent with previous studies (Babor et al., 2018; Hilton et al., 2014, 2019; Katikireddi & Hilton, 2015; Mercille, 2017; Petticrew et al., 2017; Savell et al., 2016).

In Canada, the partial government monopoly controlling alcohol distribution and sale across most jurisdictions may be impeding development of the powerful industry lobby seen in Ireland (Thomas, 2012). Nevertheless, the national trade associations and industry-funded alcohol education organizations in Canada have historically been included in public health-related policymaking bodies such as the National Alcohol Strategy Advisory Committee (Paradis, 2016), which suggests they do hold measurable political sway. The lack of advance warning given to the Canadian alcohol industry about the academic study testing AWLs, and specifically the cancer label, may have limited their ability to initiate greater inclusion of their perspectives in the news coverage, especially at the time that the new labels were launched. The industry’s repeated questioning of the legality of the AWLs is a strategy that has frequently been used to oppose other alcohol control measures (Hilton et al., 2014, 2019), but their implied threats of litigation against the Yukon government, which served to halt the labeling intervention, did not ultimately play to their favor in the news media.

This particular argument may have held less weight given no previous legal challenges had been directed at the Yukon (or neighboring Northwest Territories) government in their nearly 30-year history of applying after-market pregnancy warning labels to alcohol containers (Austen, 2018).

The pronounced inclusion of alcohol industry perspectives in the news coverage of the Ireland Bill is consistent with the broader political environment in which the Irish government’s “social partnership model” has given the alcohol industry a powerful voice in opposing public health measures (Hope, 2006; Mercille, 2016). Similar to the current findings, an earlier examination of media articles published when the Ireland Bill was first introduced in 2015 showed that coverage focused substantially on economic rather than public health considerations (Mercille, 2017); economic arguments were also frequently used to oppose minimum pricing measures in Scotland (Hilton et al., 2014). The fairly industry-friendly news coverage of the bill may also point to the relatively recent emergence of a cohesive public health lobby in Ireland (Hope, 2006). The country’s Minister for Health, however, has shown increasing evidence of the strength of this public health lobby, successfully shepherding the controversial Ireland Bill through parliament and most recently requesting that media outlets not include information provided by industry-funded social aspects public relations organizations in news stories related to alcohol (Baker, 2019).

In light of these findings, researchers and public health advocates engaging with the media may want to consider pairing the presentation of scientific evidence alongside likely tactics used in industry responses, such as those highlighted in this article, in anticipation of industry efforts to reframe public discourse and derail implementation of effective alcohol control measures (Fogarty & Chapman, 2012). This practice may also serve as an opportunity to increase awareness of the playbook of industry messaging strategies among journalists who are not as familiar with the alcohol policy arena and contribute to a shift toward more evidence based public discourse and a more public health–friendly media environment.

### Limitations

This study has several limitations. Despite some similarities, it is important to acknowledge that the contexts surrounding the two cancer warning label initiatives are quite different, with one forming part of a jurisdiction-specific academic research study and the other forming part of nationally proposed health legislation. The number of articles included in this analysis was relatively small (less than 40 articles per site) and also represents a specific time period; thus, the analysis was not able to capture the full breadth and depth of the media coverage of the Yukon Study and the Ireland Bill. Specific media platforms and search databases were used, and other sources may have yielded additional or alternative perspectives that were not identified here. Last, nuances specific to the Ireland Bill context may have been missed because the authors had more familiarity with the events surrounding the Yukon Study. Future investigations could build on the analysis of alcohol industry arguments in media coverage of AWLs by examining the public health perspectives not analyzed in the current article. In addition, detailed explorations comparing the depiction of two studies by the trade press could provide useful insight. Comparisons with media coverage of Australia’s recently mandated pregnancy warning labels are also warranted.

### Conclusions

Media coverage of the Yukon Study in Canada was largely supportive of AWLs with a cancer message, whereas coverage of the Ireland Bill was mainly opposed to the cancer labels and consistently foregrounded alcohol industry perspectives. Representatives of the alcohol industry in Canada and Ireland frequently made statements that distorted or unequivocally denied the validity of the labels’ evidence-based cancer message. Across all news coverage, industry arguments opposing the cancer label were largely consistent with the cross-industry playbook known to be used to undermine effective public health policies. Engaging with news and other media to increase awareness of the alcohol industry’s playbook of messaging strategies may enable public health researchers and advocates to generate more critical coverage of industry lobbying activities and increase public support for alcohol control measures.

## References

[B1] Alattas M. Ross C. S. Henehan E. R. Naimi T. S. 2020 Alcohol policies and alcohol-attributable cancer mortality in U.S. states *Chemico-Biological Interactions* 315 108885 doi:10.1016/j.cbi.2019.108885 31678112 10.1016/j.cbi.2019.108885

[B2] Alcoholic Beverage Health Warning Statement, 27 C.F.R § 16 2008 Retrieved from https://www.govinfo.gov/app/details/CFR-2008-title27vol1/CFR-2008-title27-vol1-part16/summary

[B3] Austen I. 2018, January 6 *Yukon gives in to liquor industry on warning label experiment* *The New York Times* Retrieved from https://www.nytimes.com/2018/01/06/world/canada/yukon-liquor-alcohol-warnings.html?partner=IFTTT

[B4] Azar D. White V. Bland S. Livingston M. Room R. Chikritzhs T. Wakefield M. 2014 ‘Something’s brewing’: The changing trends in alcohol coverage in Australian newspapers 2000–2011 *Alcohol and Alcoholism* 49 336 342 doi:10.1093/alcalc/agt139 24005573 10.1093/alcalc/agt139

[B5] Baan R. Straif K. Grosse Y. Secretan B. El Ghissassi F. Bouvard V. Cogliano V. & the WHO International Agency for Research on Cancer Monograph Working Group 2007 Carcinogenicity of alcoholic beverages *The Lancet Oncology* 8 292 293 doi:10.1016/S1470-2045(07)70099-2 10.1016/s1470-2045(07)70099-2 17431955

[B6] Babor T. F. Robaina K. Brown K. Noel J. Cremonte M. Pantani D. Pinsky I. 2018 Is the alcohol industry doing well by ‘doing good’? Findings from a content analysis of the alcohol industry’s actions to reduce harmful drinking *BMJ Open* 8 e024325 doi:10.1136/bmjopen-2018-024325 10.1136/bmjopen-2018-024325 PMC6224758 30361407

[B7] Bagnardi V. Rota M. Botteri E. Tramacere I. Islami F. Fedirko V. La Vecchia C. 2015 Alcohol consumption and site-specific cancer risk: A comprehensive dose-response meta-analysis *British Journal of Cancer* 112 580 593 doi:10.1038/bjc.2014.579 25422909 10.1038/bjc.2014.579 PMC4453639

[B8] Baker N. 2019, December 27 Simon Harris: ‘Use HSE for sound advice on alcohol *Irish Examiner* Retrieved from https://www.irishexaminer.com/breakingnews/ireland/simon-harris-use-hse-for-sound-advice-onalcohol-972336.html

[B9] Bates S. Holmes J. Gavens L. de Matos E. G. Li J. Ward B. Buykx P. 2018 Awareness of alcohol as a risk factor for cancer is associated with public support for alcohol policies *BMC Public Health* 18 688 doi:10.1186/s12889-018-5581-8 29866082 10.1186/s12889-018-5581-8 PMC5987582

[B10] Bhattacharya A. Angus C. Pryce R. Holmes J. Brennan A. Meier P. S. 2018 How dependent is the alcohol industry on heavy drinking in England? *Addiction* 113 2225 2232 doi:10.1111/add.14386 30136436 10.1111/add.14386

[B11] Burton R. Sheron N. 2018 No level of alcohol consumption improves health *The Lancet* 392 987 988 doi:10.1016/S0140-6736(18)31571-X 10.1016/S0140-6736(18)31571-X 30146328

[B12] Buykx P. Gilligan C. Ward B. Kippen R. Chapman K. 2015 Public support for alcohol policies associated with knowledge of cancer risk *International Journal on Drug Policy* 26 371 379 doi:10.1016/j.drugpo.2014.08.006 25217801 10.1016/j.drugpo.2014.08.006

[B13] Casswell S. Callinan S. Chaiyasong S. Cuong P. V. Kazantseva E. Bayandorj T. Wall M. 2016 How the alcohol industry relies on harmful use of alcohol and works to protect its profits *Drug and Alcohol Review* 35 661 664 doi:10.1111/dar.12460 27785844 10.1111/dar.12460

[B14] Chisholm D. Moro D. Bertram M. Pretorius C. Gmel G. Shield K. Rehm J. 2018 Are the “best buys” for alcohol control still valid? An update on the comparative cost-effectiveness of alcohol control strategies at the global level *Journal of Studies on Alcohol and Drugs* 79 514 522 doi:10.15288/jsad.2018.79.514 30079865

[B15] Connor J. 2017 Alcohol consumption as a cause of cancer *Addiction* 112 222 228 doi:10.1111/add.13477 27442501 10.1111/add.13477

[B16] Durrant R. Wakefield M. McLeod K. Clegg-Smith K. Chapman S. 2003 Tobacco in the news: An analysis of newspaper coverage of tobacco issues in Australia, 2001 *Tobacco Control* 12 Supplement 2 ii75 ii81 doi:10.1136/tc.12.suppl_2.ii75 12878777 10.1136/tc.12.suppl_2.ii75 PMC1766104

[B17] Elo S. Kyngäs H. 2008 The qualitative content analysis process *Journal of Advanced Nursing* 62 107 115 doi:10.1111/j.1365-2648.2007.04569.x 18352969 10.1111/j.1365-2648.2007.04569.x

[B18] Fogarty A. S. Chapman S. 2012 Advocates, interest groups and Australian news coverage of alcohol advertising restrictions: Content and framing analysis *BMC Public Health* 12 727 doi:10.1186/1471-2458-12-727 22938674 10.1186/1471-2458-12-727 PMC3495017

[B19] Foster S. Gmel G. Mohler-Kuo M. 2019 Light and heavy drinking in jurisdictions with different alcohol policy environments *International Journal of Drug Policy* 65 86 96 doi:10.1016/j.drugpo.2019.01.014 30711804 10.1016/j.drugpo.2019.01.014

[B20] Greenfield T. K. 1997 Warning labels: Evidence of harm reduction from long-term American surveys Plant M. Single E. Stockwell T. *Alcohol: Minimizing the harm (pp. 105–125)* London, England Free Association Books

[B21] Hilton S. Buckton C. H. Patterson C. Katikireddi S. V. Lloyd-Williams F. Hyseni L. Capewell S. 2019 Following in the footsteps of tobacco and alcohol? Stakeholder discourse in UK newspaper coverage of the Soft Drinks Industry Levy *Public Health Nutrition* 22 2317 2328 doi:10.1017/S1368980019000739 31111808 10.1017/S1368980019000739 PMC6642695

[B22] Hilton S. Wood K. Patterson C. Katikireddi S. V. 2014 Implications for alcohol minimum unit pricing advocacy: What can we learn for public health from UK newsprint coverage of key claim-makers in the policy debate? *Social Science & Medicine* 102 157 164 doi:10.1016/j.socscimed.2013.11.041 24565153 10.1016/j.socscimed.2013.11.041 PMC3991846

[B23] Hobin E. Vallance K. Zuo F. Stockwell T. Rosella L. Simniceanu A. Hammond D. 2018 Testing the efficacy of alcohol labels with standard drink information and national drinking guidelines on consumers’ ability to estimate alcohol consumption *Alcohol and Alcoholism* 53 3 11 doi:10.1093/alcalc/agx052 29016708 10.1093/alcalc/agx052

[B24] Hobin E. Weerasinghe A. Vallance K. Hammond D. McGavock J. Greenfield T. K. Stockwell T. 2020 Testing alcohol labels as a tool to communicate cancer risk to drinkers: A real-world quasi-experimental study *Journal of Studies on Alcohol and Drugs* 81 249 261 doi:10.15288/jsad.2020.81.249 32359056 10.15288/jsad.2020.81.249 PMC7201213

[B25] Hope A. 2006 The influence of the alcohol industry on alcohol policy in Ireland *Nordic Studies on Alcohol and Drugs* 23 467 481 doi:10.1177/145507250602300612

[B26] International Agency for Research on Cancer 2010 *IARC monographs on the evaluation of carcinogenic risks to humans. Volume 96: Alcohol consumption and ethyl carbamate* Lyon, France Author Retrieved from https://monographs.iarc.fr/wp-content/uploads/2018/06/mono96.pdf PMC4781168 21735939

[B27] Joannou A. 2018, February 16 Yukon’s alcohol label study back on but without a cancer warning *Yukon News* Retrieved from https://www.yukon-news.com/news/yukons-alcohol-label-study-back-on-but-withouta-cancer-warning/

[B28] Karriker-Jaffe K. J. Room R. Giesbrecht N. Greenfield T. K. 2018 Alcohol’s harm to others: Opportunities and challenges in a public health framework *Journal of Studies on Alcohol and Drugs* 79 239 243 doi:10.15288/jsad.2018.79.239 29553351 10.15288/jsad.2018.79.239 PMC6019779

[B29] Katikireddi S. V. Hilton S. 2015 How did policy actors use mass media to influence the Scottish alcohol minimum unit pricing debate? Comparative analysis of newspapers, evidence submissions and interviews *Drugs: Education, Prevention and Policy* 22 125 134 doi:10.3109/09687637.2014.977228 10.3109/09687637.2014.977228 PMC4438355 26045639

[B30] Klein W. M. P. Jacobsen P. B. Helzlsouer K. J. 2019 Alcohol and cancer risk: Clinical and research implications *JAMA* 323 23 24 doi:10.1001/jama.2019.19133 10.1001/jama.2019.19133 31834355

[B31] Lemmens P. H. Vaeth P. A. Greenfield T. K. 1999 Coverage of beverage alcohol issues in the print media in the United States, 1985–1991 *American Journal of Public Health* 89 1555 1560 doi:10.2105/AJPH.89.10.1555 10511839 10.2105/ajph.89.10.1555 PMC1508794

[B32] Li J. Lovatt M. Eadie D. Dobbie F. Meier P. Holmes J. MacKintosh A. M. 2017 Public attitudes towards alcohol control policies in Scotland and England: Results from a mixed-methods study *Social Science & Medicine* 177 177 189 doi:10.1016/j.socscimed.2017.01.037 28171817 10.1016/j.socscimed.2017.01.037 PMC5341733

[B33] Lim A. W. Y. van Schalkwyk M. C. I. Maani Hessari N. Petticrew M. P. 2019 Pregnancy, fertility, breastfeeding, and alcohol consumption: An analysis of framing and completeness of information disseminated by alcohol industry–funded organizations *Journal of Studies on Alcohol and Drugs* 80 524 533 doi:10.15288/jsad.2019.80.524 31603753 10.15288/jsad.2019.80.524 PMC6811724

[B34] Maani Hessari N. Petticrew M. 2018 What does the alcohol industry mean by ‘Responsible drinking’? A comparative analysis *Journal of Public Health* 40 90 97 doi:10.1093/pubmed/fdx040 28398571 10.1093/pubmed/fdx040

[B35] Maani Hessari N. van Schalkwyk M. C. Thomas S. Petticrew M. 2019 Alcohol industry CSR organisations: What can their Twitter activity tell us about their independence and their priorities? A comparative analysis *International Journal of Environmental Research and Public Health* 16 892 doi:10.3390/ijerph16050892 10.3390/ijerph16050892 PMC6427731 30871025

[B36] Macnamara J. 2005 Media content analysis: Its uses; benefits and best practice methodology *Asia Pacific Public Relations Journal* 6 1 34 Retrieved from http://hdl.handle.net/10453/10102

[B37] Manthey J. Shield K. D. Rylett M. Hasan O. S. M. Probst C. Rehm J. 2019 Global alcohol exposure between 1990 and 2017 and forecasts until 2030: A modelling study *The Lancet* 393 2493 2502 doi:10.1016/S0140-6736(18)32744-2 10.1016/S0140-6736(18)32744-2 31076174

[B38] Martin N. Buykx P. Shevills C. Sullivan C. Clark L. NewburyBirch D. 2018 Population level effects of a mass media alcohol and breast cancer campaign: A cross-sectional pre-intervention and post-intervention evaluation *Alcohol and Alcoholism* 53 31 38 doi:10.1093/alcalc/agx071 29155922 10.1093/alcalc/agx071

[B39] Maynard O. Blackwell A. Munafò M. Attwood A. 2018 *Know your limits: Labelling interventions to reduce alcohol consumption* Alcohol Research UK. Retrieved from https://s3.eu-west-2.amazonaws.com/files.alcoholchange.org.uk/documents/FinalReport_0150.pdf?mtime=20181110145642

[B40] McCambridge J. Mialon M. Hawkins B. 2018 Alcohol industry involvement in policymaking: A systematic review *Addiction* 113 1571 1584 doi:10.1111/add.14216 10.1111/add.14216 PMC6100095 29542202

[B41] McCombs M. Shaw D. 1972 The agenda-setting functions of mass media *Public Opinion Quarterly* 36 176 187 doi:10.1086/267990

[B42] McCombs M. Shaw D. 1993 The evolution of agenda-setting research: Twenty-five years in the marketplace of ideas *Journal of Communication* 43 58 67 doi:10.1111/j.1460-2466.1993.tb01262.x

[B43] McHugh M. L. 2012 Interrater reliability: The kappa statistic *Biochemia Medica* 22 276 282 doi:10.11613/BM.2012.031 23092060 PMC3900052

[B44] Mercille J. 2016 Neoliberalism and the alcohol industry in Ireland *Space and Polity* 20 59 74 doi:10.1080/13562576.2015.1072914

[B45] Mercille J. 2017 Media coverage of alcohol issues: A critical political economy framework—A case study from Ireland *International Journal of Environmental Research and Public Health* 14 650 doi:10.3390/ijerph14060650 10.3390/ijerph14060650 PMC5486336 28621753

[B46] Miller C. L. Brownbill A. L. Dono J. Ettridge K. 2018 Presenting a strong and united front to tobacco industry interference: A content analysis of Australian newspaper coverage of tobacco plain packaging 2008-2014 *BMJ Open* 8 e023485 doi:10.1136/bmjopen-2018-023485 10.1136/bmjopen-2018-023485 PMC6144411 30224400

[B47] Miller E. R. Ramsey I. J. Baratiny G. Y. Olver I. N. 2016 Message on a bottle: Are alcohol warning labels about cancer appropriate? *BMC Public Health* 16 139 doi:10.1186/s12889-016-2812-8 26864239 10.1186/s12889-016-2812-8 PMC4750299

[B48] Moher D. Liberati A. Tetzlaff J. Altman D. G. & the PRISMA Group 2009 Preferred reporting items for systematic reviews and meta-analyses: The PRISMA statement *PLoS Medicine* 6 e1000097 doi:10.1371/journal.pmed.1000097 10.1371/journal.pmed.1000097 PMC2707599 19621072

[B49] Moskalewicz J. Wieczorek Ł. Karlsson T. Österberg E. 2013 Social support for alcohol policy: Literature review *Drugs: Education, Prevention and Policy* 20 361 374 doi:10.3109/09687637.2012.687794

[B50] Murray F. 2017 Ireland’s Public Health Bill: Crucial to reduce alcohol harm *The Lancet* 390 2222 2223 doi:10.1016/S0140-6736(17)32759-9 10.1016/S0140-6736(17)32759-9 29103655

[B51] Paradis C. 2016 Canada’s National Alcohol Strategy: It’s time to assess progress *Canadian Journal of Program Evaluation* 31 232 241 doi:10.3138/cjpe.276

[B52] Pechey R. Burge P. Mentzakis E. Suhrcke M. Marteau T. M. 2014 Public acceptability of population-level interventions to reduce alcohol consumption: A discrete choice experiment *Social Science & Medicine* 113 104 109 doi:10.1016/j.socscimed.2014.05.010 24858928 10.1016/j.socscimed.2014.05.010 PMC4065329

[B53] Petticrew M. Katikireddi S. V. Knai C. Cassidy R. Maani Hessari N. Thomas J. Weishaar H. 2017 ‘Nothing can be done until everything is done’: The use of complexity arguments by food, beverage, alcohol and gambling industries *Journal of Epidemiology and Community Health* 71 1078 1083 doi:10.1136/jech-2017-209710 28978619 10.1136/jech-2017-209710 PMC5847098

[B54] Petticrew M. Maani Hessari N. Knai C. Weiderpass E. 2018a How alcohol industry organisations mislead the public about alcohol and cancer *Drug and Alcohol Review* 37 293 303 doi:10.1111/dar.12596 28881410 10.1111/dar.12596

[B55] Petticrew M. Maani Hessari N. Knai C. Weiderpass E. 2018b The strategies of alcohol industry SAPROs: Inaccurate information, misleading language and the use of confounders to downplay and misrepresent the risk of cancer *Drug and Alcohol Review* 37 313 315 doi:10.1111/dar.12677 29446154 10.1111/dar.12677 PMC5873370

[B56] Pettigrew S. Hafekost C. Jongenelis M. Pierce H. Chikritzhs T. Stafford J. 2018 Behind closed doors: The priorities of the alcohol industry as communicated in a trade magazine *Frontiers in Public Health* 6 217 doi:10.3389/fpubh.2018.00217 30109222 10.3389/fpubh.2018.00217 PMC6079248

[B57] Pettigrew S. Jongenelis M. Chikritzhs T. Slevin T. Pratt I. S. Glance D. Liang W. 2014 Developing cancer warning statements for alcoholic beverages *BMC Public Health* 14 786 doi:10.1186/1471-2458-14-786 25087010 10.1186/1471-2458-14-786 PMC4133604

[B58] Praud D. Rota M. Rehm J. Shield K. Zato ski W. Hashibe M. Boffetta P. 2016 Cancer incidence and mortality attributable to alcohol consumption *International Journal of Cancer* 138 1380 1387 doi:10.1002/ijc.29890 26455822 10.1002/ijc.29890

[B59] Savell E. Fooks G. Gilmore A. B. 2016 How does the alcohol industry attempt to influence marketing regulations? A systematic review *Addiction* 111 18 32 doi:10.1111/add.13048 26173765 10.1111/add.13048 PMC4681589

[B60] Thomas G. 2012 *Analysis of beverage alcohol sales in Canada* Ottawa, Ontario, Canada Canadian Centre on Substance Use and Addiction. Retrieved from https://ccsa.ca/sites/default/files/2019-04/CCSA-Analysis-Alcohol-Sales-Policies-Canada-2012-en.pdf

[B61] Vallance K. Romanovska I. Stockwell T. Hammond D. Rosella L. Hobin E. 2018 “We Have a Right to Know”: Exploring consumer opinions on content, design and acceptability of enhanced alcohol labels *Alcohol and Alcoholism* 53 20 25 doi:10.1093/alcalc/agx068 29016716 10.1093/alcalc/agx068

[B62] Vallance K. Stockwell T. Hammond D. Shokar S. Schoueri-Mychasiw N. Greenfield T. Hobin E. 2020 Testing the effectiveness of enhanced alcohol warning labels and modifications resulting from alcohol industry interference in Yukon, Canada: Protocol for a quasi-experimental study *JMIR Research Protocols* 9 e16320 doi:10.2196/16320 31922493 10.2196/16320 PMC6996737

[B63] Weerasinghe A. Schoueri-Mychasiw N. Vallance K. Stockwell T. Hammond D. McGavock J. Hobin E. 2020 Improving knowledge that alcohol can cause cancer is associated with consumer support for alcohol policies: Findings from a real-world alcohol labelling study *International Journal of Environmental Research and Public Health* 17 398 doi:10.3390/ijerph17020398 10.3390/ijerph17020398 PMC7014334 31936173

[B64] Wilt J 2018, May 21 Alcohol-industry officials lobbied Yukon to halt warning-label study, emails show *The Globe and Mail* Retrieved from https://www.theglobeandmail.com/canada/article-alcohol-industryofficials-lobbied-yukon-to-halt-warning-label-study

[B65] World Health Organization 2013 *Global action plan for the prevention and control of noncommunicable diseases 2013–2020* Geneva, Switzerland Author Retrieved from https://www.who.int/iris/bitstream/10665/94384/1/9789241506236_eng.pdf?ua=1

[B66] World Health Organization 2018 *Global status report on alcohol and health 2018* Geneva, Switzerland Author Retrieved from https://www.who.int/substance_abuse/publications/global_alcohol_report/en

